# Quantifying emotion-dependent brain–eye interactions during audiovisual emotional stimulation

**DOI:** 10.3389/fnsys.2026.1744042

**Published:** 2026-04-15

**Authors:** Feryal A. Alskafi, Ahsan H. Khandoker, Faezeh Marzbanrad, Herbert F. Jelinek

**Affiliations:** 1 Department of Biomedical Engineering and Biotechnology, Khalifa University, Abu Dhabi, United Arab Emirates; 2 Healthcare Engineering Innovation Group (HEIG), Khalifa University, Abu Dhabi, United Arab Emirates; 3 Department of Electrical and Computer Systems Engineering, Monash University, Clayton, VIC, Australia; 4 Department of Medical Sciences, Khalifa University, Abu Dhabi, United Arab Emirates

**Keywords:** affective neuroscience, brain–body interactions, cognition, EEG, EEG–EOG coupling, emotions, network physiology, time delay stability

## Abstract

**Purpose:**

Electrooculography (EOG) provides a noninvasive measure of eye movements linked to affective processing, yet it is mainly used for artifact correction of electroencephalography (EEG) signals rather than analyzed as a physiological signal in its own right. EEG–EOG coupling has therefore not been well-established. This study aimed to determine whether emotion-specific changes in arousal and valence are reflected in directional and frequency-specific interactions between EEG rhythms and EOG signals.

**Methods:**

The DEAP dataset with 32 participants, where each viewed 40 1-min music videos and rated their arousal/valence, was used (1,280 samples). EEG from eight electrodes was filtered into theta, alpha, beta, and gamma frequency bands, while horizontal and vertical EOG were also preprocessed. EOG complexity was assessed using sample, fuzzy, and permutation entropy. EEG–EOG coupling was assessed with the controlled time delay stability (CTDS) framework, which evaluates stability of partial cross-correlation delays.

**Results:**

Entropy analysis showed emotion-related differences in horizontal and vertical EOG complexity (*p* < 0.005). EEG–EOG coupling varied with emotion, with the strongest effects at sensorimotor and frontal sites, primarily within the gamma band. Directional EOG-to-EEG coupling predominating at frontal, sensorimotor, and occipital sites. Differences were most pronounced when arousal and valence varied independently or in opposite directions, with fewer effects during parallel shifts.

**Conclusion:**

Emotional states are mirrored by frequency- and channel-specific shifts in EEG–EOG interactions, a core component of the affective behavioral network. These results clarify the directional dynamics linking eye movement and cortical activity, revealing a structured, context-sensitive neural architecture for affective processing.

## 1 Introduction

Emotions are shaped by complex brain-body interactions that coordinate physiological and behavioral responses, extending beyond traditional brain-centric models (Barooah, 2019). Traditional affective models have primarily focused on identifying the neural substrates of emotion and their roles in generating and regulating emotional states (Dalgleish et al., 2009). However, recent advances in affective neuroscience suggest that emotions and cognition are the result of salient, coordinated brain–body responses (D'Hondt et al., 2010; Criscuolo et al., 2022). Contemporary frameworks move beyond brain-centric models and emphasize the embodied nature of emotional experience (Davey et al., 2021; Alskafi et al., 2022). Emotional, cognitive, and conscious processing are increasingly understood as emerging from dynamic brain-body interactions (Criscuolo et al., 2022; Signorelli et al., 2022; Cai et al., 2024; Younis et al., 2026). Bodily states and actions actively influence how emotional information is perceived, processed, and regulated (Critchley and Harrison, 2013). This perspective supports the growing view that expressed emotions are not restricted to cortical and subcortical processing but arise through continuous, bidirectional brain-body interactions.

Among the body systems, the visual system plays a particularly important role in reflecting, perceiving, and shaping emotional experience (Kragel et al., 2019; Zamuner, 2012). Eye movements not only determine which aspects of a scene are sampled but also provide a continuous behavioral readout of ongoing cognitive and motivational states (König et al., 2016). During natural viewing, gaze trajectories are shaped by internal goals, task demands, and predictions about forthcoming sensory input rather than by stimulus properties alone (Hayhoe and Ballard, 2005; König et al., 2016). This embodied perspective suggests that oculomotor behavior is functionally coupled to cortical mechanisms of attention, decision-making, and affective appraisal.

Recent neuroimaging evidence indicates that affective information engages not only canonical emotion-related regions such as the amygdala and prefrontal cortex but also occipital and retinotopic visual areas, with affective scene representations emerging dynamically over time through recurrent interactions across distributed cortical networks (Bo et al., 2022; Phan et al., 2004; Celeghin et al., 2017; Nanni et al., 2018; Bo et al., 2021). These findings highlight that emotional visual processing is temporally structured and supported by bidirectional communication between sensory and higher-order systems, implying that eye-movement dynamics are an integral component of affective processing rather than a passive byproduct of stimulus viewing.

While imaging techniques have been instrumental in identifying the spatial architecture of emotional processing, they remain limited in their temporal resolution, expensive, and often impractical for use in naturalistic or everyday settings. In contrast, electroencephalography (EEG) and electrooculography (EOG) provide millisecond-level resolution of cortical and oculomotor activity (Chang, 2019; Rahman et al., 2021), allowing direct quantification of dynamic brain–eye interactions and thereby providing a more holistic view of emotional experience.

In parallel with advances in mechanistic signal analysis, artificial intelligence (AI)–based approaches to EEG have rapidly evolved. Recent work has leveraged deep neural networks, attention-based architectures, and unified representation learning frameworks to model complex spatial–temporal EEG dynamics and enable multimodal integration across neural signals (Chen et al., 2025; Alskafi et al., 2023b; Cao, 2020). These approaches have demonstrated strong performance in cognitive and affective state decoding, as well as cross-modal neural signal modeling (Li et al., 2025). Such data-driven frameworks are increasingly shaping the landscape of EEG analysis by emphasizing large-scale pattern learning and end-to-end optimization. However, while AI models often prioritize predictive accuracy, they do not always explicitly characterize the directional and mechanistic interactions between physiological subsystems. The present study complements these developments by adopting a framework that focuses on structured, frequency-specific brain–eye coupling during affective processing.

Importantly, examining the coupling between cortical rhythms and oculomotor activity allows investigation not merely of eye behavior or brain activity in isolation, but of their coordinated and temporally structured interaction during emotional processing. Accordingly, brain–eye coupling represents a mechanistically grounded sensory–motor loop through which ascending visual signals convey emotionally salient information and descending cortical signals modulate gaze behavior in line with contextual demands. Quantifying EEG–EOG interactions therefore targets a mechanistically grounded system directly implicated in dynamic affective processing.

To investigate these interactions, various analytical approaches have been developed. Traditional correlation analyses are commonly used to assess shared activity or frequency content between physiological signals but are limited to linear and undirected relationships (Janse et al., 2021). More advanced methods, such as Granger causality, transfer entropy, and dynamic causal modeling, aim to estimate directionality and information flow between signals (Friston et al., 2013). However, these approaches often rely on prior assumptions such as signal stationarity and the availability of long time series, which limit their applicability to transient emotional states (Maziarz, 2015). To overcome these limitations, Time Delay Stability (TDS) has been proposed as a general framework for identifying transient synchronous bursts, which are considered a hallmark of physiological network communication. TDS is well suited for heterogeneous, non-stationary signals with time-varying coupling and has demonstrated greater reliability than traditional approaches (Bashan et al., 2012; Bartsch et al., 2015; Ivanov et al., 2021; Rizzo et al., 2022). Building on this, the Controlled Time Delay Stability (CTDS) framework extends TDS by quantifying pairwise, directional coupling while accounting for the influence of indirect interactions (Marzbanrad et al., 2020; Alskafi et al., 2023a, 2024, 2023b).

Given its ability to capture dynamic and directional relationships while controlling for indirect influences, the CTDS framework provides a principled approach to quantifying coordinated physiological network activity. In this study, we applied CTDS to simultaneously recorded EEG and EOG signals during emotionally evocative audiovisual stimuli to examine brain–eye interactions across affective states. Rather than treating ocular activity as a confound or byproduct of visual stimulation, we test the hypothesis that emotional states are reflected through the reorganization of the directional flow of information between cortical rhythms and oculomotor dynamics. Specifically, we predict that distinct combinations of arousal and valence will be associated with frequency-specific and region-specific patterns of coupling, reflecting shifts in bottom-up sensory drive and top-down modulatory control. Demonstrating such structured, affect-dependent reconfiguration would support the view that brain–eye interactions constitute an integral component of the affective network architecture rather than a secondary correlate of stimulus processing.

## 2 Methods

### 2.1 Data

This study analyzed physiological recordings from the DEAP dataset, a multimodal database created to investigate emotional responses to audiovisual stimuli (Koelstra et al., 2011). The dataset comprises 32 healthy, predominantly European participants (see Supplementary Table S1). Each participant viewed forty one-minute music video excerpts selected to elicit a wide range of emotions, yielding 1280 samples (32 participants × 40 videos). The 40 selected music-video excerpts and their valence and arousal ratings from the online annotation phase (mean ± SD) are provided in Supplementary Table S2. No further exclusion criteria were reported by the authors, and all participants and trials were included in the present analysis (Koelstra et al., 2011). The videos were played on two-thirds of a 17-inch screen to control the range of eye movements. During every clip, EEG and other peripheral signals were recorded, and viewers rated their felt arousal and valence on a nine-point self-assessment manikin scale. Scores were binarized using a midpoint split at 4.5: values ≤ 4.5 were treated as low and values ≥4.5 as high, for both arousal and valence. This produces labels corresponding to the four quadrants in Russell's circumplex model of affect (Russell, 1980): low arousal–low valence (LALV), low arousal–high valence (LAHV), high arousal–low valence (HALV), and high arousal–high valence (HAHV). Each of the forty trials per participant was then categorized into the corresponding quadrant.

EEG signals were originally recorded from 32 active AgCl electrodes positioned according to the international 10–20 system using a Biosemi ActiveTwo system (BioSemi B.V., Netherlands) at a sampling rate of 512 Hz (Koelstra et al., 2011). The preprocessed EEG signals provided with the publicly available DEAP dataset were downsampled to 128 Hz, band-pass filtered, re-referenced to the common average, and cleaned of ocular artifacts using independent component analysis (ICA) prior to public release (Koelstra et al., 2011). Although the original publication does not report the exact number of independent components removed per participant, components reflecting ocular activity were identified and excluded during dataset preprocessing. The preprocessed signals were used without additional artifact rejection beyond this ICA-based correction to ensure methodological reproducibility while relying on the validated preprocessing pipeline described in (Koelstra et al. 2011). Eight EEG channels were selected to provide representative bilateral coverage of major cortical regions implicated in affective and audiovisual processing (Fp1, Fp2, C3, C4, T7, T8, O1, and O2). These electrodes correspond to frontal–prefrontal regions associated with attentional control and emotional appraisal (Fp1/Fp2), central sensorimotor areas involved in visuomotor integration (C3/C4), temporal regions supporting auditory and affective processing (T7/T8), and occipital sites responsible for visual perception (O1/O2) (Chapin and Russell-Chapin, 2013). Because participants viewed emotionally evocative audiovisual stimuli, this regional distribution captures cortical systems jointly engaged in attention, perception, and sensorimotor coordination. Importantly, CTDS involves higher-order partial cross-correlation conditioning on all remaining signals. Including all 32 electrodes would substantially increase model dimensionality, reduce statistical stability, and inflate indirect dependencies. Selecting representative nodes from each major cortical region allows preservation of large-scale spatial organization while maintaining computational tractability and interpretability of directional interactions. This regionally distributed and symmetric montage therefore balances theoretical coverage with methodological robustness. Each selected EEG channel was then analyzed across four distinct EEG rhythms: theta (4–7 Hz), alpha (8–12 Hz), beta (13–29 Hz), and gamma (30–45 Hz).

EOG was recorded using two pairs of electrodes: one placed above and below the left eye (vertical EOG) and another at the outer canthi (horizontal EOG). Vertical (vEOG) and horizontal (hEOG) signals reflect distinct oculomotor axes involved in gaze control. vEOG primarily captures vertical eye movements and blink-related activity, whereas hEOG reflects lateral saccades and horizontal gaze shifts (Kowler, 2011). Because eye movements are closely integrated with attentional selection and task demands (Kowler, 2011), emotional audiovisual stimuli may differentially modulate vertical and horizontal scanning dynamics. The signals were provided in preprocessed form within the DEAP dataset, including downsampling to 128 Hz. In the present study, baseline drift was removed by mean-centering each trial, and a bandpass filter (0.1–15 Hz) was applied using a 6th-order finite impulse response (FIR) filter designed via least-squares optimization to isolate oculomotor activity and eliminate high-frequency components such as saccadic spike potentials (Banerjee et al., 2013).

### 2.2 Entropy measures

The complexity of the EOG signal was assessed using three entropy measures: sample entropy (SampEn), fuzzy entropy (FuzzyEn), and permutation entropy (PermEn), implemented using the *EntropyHub* toolbox (Flood and Grimm, 2021). Each method captures a different aspect of signal complexity. Entropy quantifies the degree of irregularity or unpredictability in a time series. Higher entropy values indicate more irregular, less predictable, and more variable eye-movement dynamics, whereas lower entropy values reflect more regular, stereotyped, or repetitive patterns of ocular activity. SampEn estimates the negative natural logarithm of the conditional probability that two sequences similar for *m* points remain similar at *m*+1 (Richman and Moorman, 2000). Lower SampEn values therefore indicate greater self-similarity and regularity in the signal, while higher values reflect increased unpredictability. FuzzyEn extends this approach by incorporating fuzzy sets to evaluate time series regularity (Chen et al., 2007). By using graded similarity functions rather than a strict threshold, FuzzyEn provides a noise-robust estimate of signal regularity, with higher values corresponding to greater complexity. PermEn is an ordinal-based, non-parametric measure of temporal dependence structure (Bandt and Pompe, 2002; Zanin et al., 2012; Huang et al., 2022). It quantifies the diversity of ordinal patterns in the signal; higher PermEn values indicate a broader distribution of rank-order patterns and thus greater temporal complexity. In the context of EOG signals, higher entropy reflects more variable or exploratory eye-movement dynamics, whereas lower entropy suggests more constrained or stereotyped oculomotor behavior. These measures therefore provide complementary characterizations of ocular complexity during emotional audiovisual stimulation.

All entropy measures were calculated with an embedding dimension of *m* = 4, selected to provide a reliable characterization of the temporal structure of the EOG signal. Remaining parameters were set to default values: time delay = 1; for SampEn, tolerance radius = 0.2 × standard deviation of the signal; for FuzzyEn, fuzzy function = “default” with parameters [0.2, 2] and natural logarithm; and for PermEn, base 2 logarithm was used. Permutation entropy values are therefore reported in log_2_ units, with a theoretical maximum of log_2_(*m*!) for embedding dimension *m* = 4 (Flood and Grimm, 2021).

### 2.3 Controlled time delay stability

**Figure 1:**
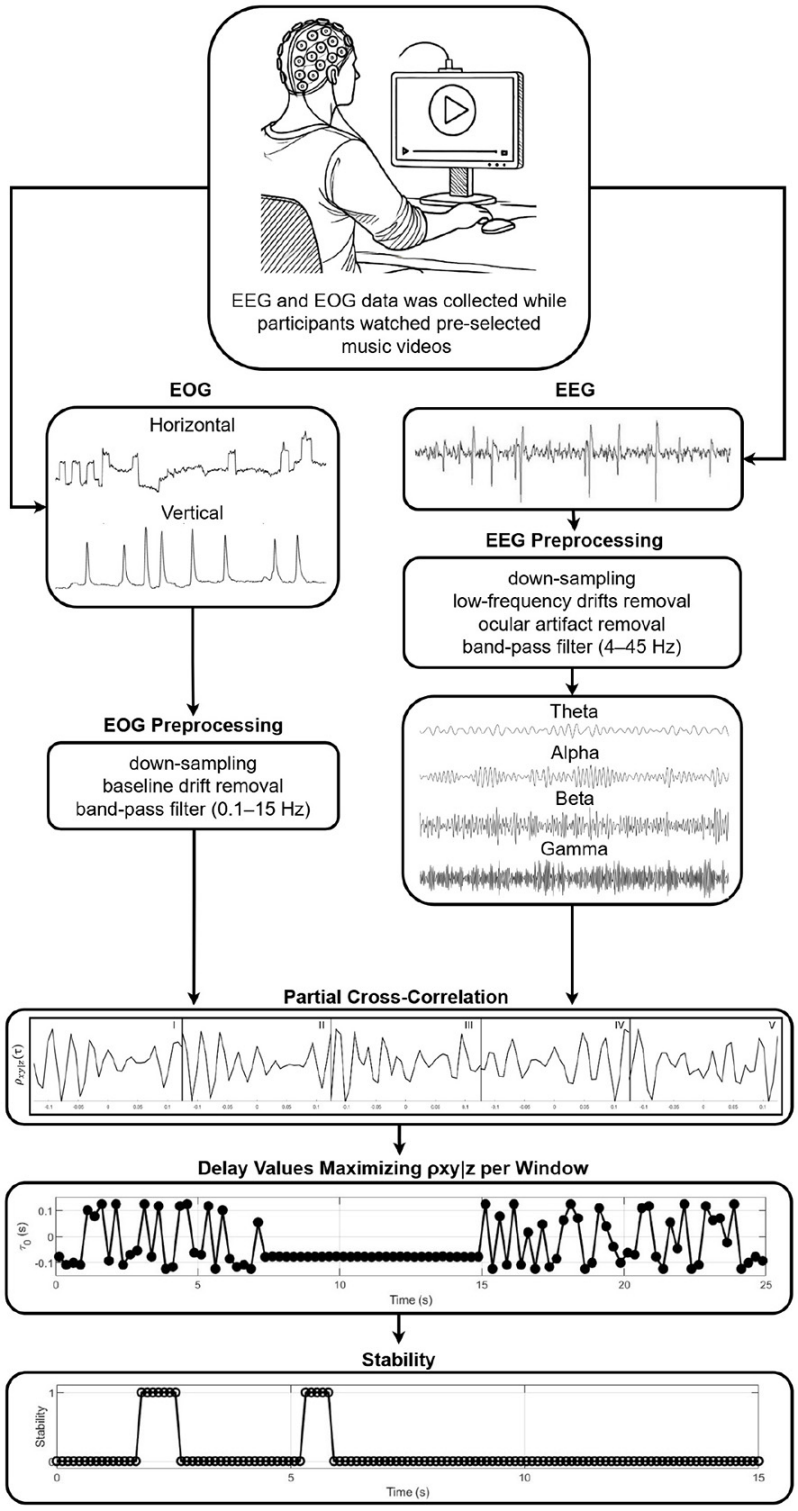
A schematic of the brain-body interactions quantification framework used in this study. EEG and EOG signals were first preprocessed and then analyzed in successive window boundaries. Within a window, ρ_*xy*|*z*_ captures the synchronous bursts between the first signal *S*_*x*_ and the second signal *S*_*y*_ in clear partial cross-correlation peaks while controlling for a third signal *S*_*z*_. The delay $\tau_{0}^{v}$ that maximizes the function ρ_*xy*|_*z*__1_, *z*_2_, ..., *z*_*N*__(τ) in each window is identified. Stability is then defined by several consecutive segments in which $\tau_{0}^{v}$ remains within ±1 delay unit of a reference delay τ_0_, and coupling strength between two signals is then defined using this framework as the percentage of the total duration during which stable delays are observed (%CTDS). Longer durations of stability reflect stronger coupling.

The CTDS framework was used to quantify the directional coupling between the physiological signals (Figure 1). This framework combines higher-order partial cross-correlation with time delay stability analysis to identify directed interactions in non-stationary systems (Bashan et al., 2012; Marzbanrad et al., 2020). All signals were z-scored prior to analysis to eliminate amplitude-related bias in the coupling estimates (Zitouni et al., 2022). CTDS was implemented separately for each EEG channel. For a given electrode (e.g., Fp1), interactions were evaluated among its four frequency bands (theta, alpha, beta, gamma) and the two EOG signals (vEOG and hEOG). Thus, each channel-specific model included six signals in total.

The relationship between two physiological signals *x* and *y*, while controlling for the influence of *N* additional physiological signals *z*_1_, *z*_2_, ..., *z*_*N*_, was estimated recursively using *N*th-order partial cross-correlation, as defined in Equation 1.


$$\begin{array}{l} \rho_{xy|z_{1},z_{2},...,z_{N}}\left(\tau \right)=\\ \frac{\rho_{xy|z_{1},z_{2},...,z_{N}}\left(\tau \right)-\rho_{xz_{N}|z_{1},z_{2},...,z_{N-1}}.\rho_{z_{N}y|z_{1},z_{2},...,z_{N-1}}\left(\tau \right)}{\sqrt{1-\rho_{xz_{N}|z_{1},z_{2},...,z_{N-1}}^{2}}\sqrt{1-\rho_{z_{N}y|z_{1},z_{2},...,z_{N-1}}\left(\tau \right)}} \end{array}$$ (1)

For each directed interaction between an EEG band and an EOG signal (e.g., Fp1) γ → hEOG, the control variables consisted of the remaining three frequency bands from the same EEG channel and the alternate EOG axis. Therefore, *N* = 4 control signals were included in each computation. This conditioning isolates direct band-specific brain–eye interactions while accounting for within-channel cross-frequency dependencies and inter-axis ocular influences. This computation was performed for overlapping segments *v* of length *l* = 250*milliseconds*(*ms*), with 50% overlap. The 250 *ms* window was selected to align with the time scales of the four EEG rhythms studied and accommodate at least one full theta cycle and several alpha, beta, and gamma cycles, while remaining sufficiently brief to capture the emotion-related neural activity that unfolds over hundreds of milliseconds (Khanna et al., 2015; Liu et al., 2023; Hu et al., 2023), as well as the oculomotor events that generally occur within intervals of ten to several hundred milliseconds (Toivanen et al., 2015). This duration therefore provides adequate temporal resolution, while the 50% overlap maintains statistical stability in the coupling estimates.

For each segment *v*, the time delay $\tau_{0}^{v}$ that maximized ρ_*xy*|_*z*__1_, *z*_2_, ..., *z*_*N*__(τ) was identified Equation 2.


$$\begin{array}{l} \tau_{0}^{v}={\text{argmax}}_{\tau }|\rho_{xy|z_{1},z_{2},\ldots ,z_{N}}\left(\tau \right)| \end{array}$$ (2)

As defined in (Bashan et al. 2012), for a given delay τ_0_ within the delay time series $\tau_{0}^{v}$, stability is established when the delay τ_0_ remains within the interval [τ_0_−1, τ_0_+1] for at least 0.8*H consecutive segments within a sliding window of *H* segments. Following previous work (Bashan et al., 2012; Marzbanrad et al., 2020; Cai et al., 2024), *H* was set to five segments in this study (*H* = 5), meaning that a delay was considered stable if it remained approximately constant for at least four out of five consecutive segments (Equation 3).


$$\begin{array}{l} Stability\left(\tau_{0}\right)=\overset{N-L+1}{\underset{v=1}{\sum }}I\left(|\tau_{0}^{v}-\tau_{0}\right)|\leq 1\geq 4, \end{array}$$ (3)

where *I*(.) is the indicator function.

The strength of coupling between signals was defined as the percentage of time during which stable delays were observed (Equation 4).


$$\begin{array}{l} \%\text{CTDS}=\frac{\text{Number of Stable Points}}{L-l+1} \end{array}$$ (4)

where *L* is the total signal duration. Higher %CTDS values indicate stronger physiological coupling (Marzbanrad et al., 2020).

To compute group-averaged interaction strength, outliers caused by artifacts or individual variability were removed. For each link, the distribution and standard deviation of %CTDS values across all samples were calculated, and any value exceeding the mean by more than two standard deviations was excluded. The average was then recomputed using the remaining samples. This procedure was applied to all interactions to ensure consistent outlier removal (Ivanov et al., 2021).

### 2.4 Statistical analysis

The nonparametric Kruskal–Wallis test was used to assess significant differences in the coupling strength between emotions for each interaction studied (Ostertagova et al., 2014). Statistical analyses were conducted at the trial level, with trials assigned to affective categories based on participants' valence–arousal ratings. Because participants contributed unequal numbers of trials to each category, the resulting design was unbalanced. We note that this approach does not explicitly model within-subject dependence and therefore provides an approximate comparison across conditions. All statistical analyses were performed at a 95% significance level. When the omnibus test was significant (*p* < 0.05), *post hoc* pairwise comparisons were conducted using the Dunn–Šidák method, which adjusts *p*-values to control the family-wise error rate (FWER) across multiple comparisons (Agbangba et al., 2024). FWER control was implemented separately for each electrode–interaction pair, treating the six pairwise affect comparisons as a single comparison family. No additional FWER control was applied across electrodes or interaction types, as the analyses were designed to support interaction-specific rather than global network-level inferences.

## 3 Results

The primary analysis in this study aimed to quantify the directional interactions between ocular and cortical activity by computing the %CTDS between hEOG and vEOG signals and EEG frequency bands across the four affective states consisting of high arousal–high valence (HAHV), high arousal–low valence (HALV), low arousal–high valence (LAHV), and low arousal–low valence (LALV). To confirm that the EOG recordings reflected structured eye-movement behavior rather than random fluctuations or purely visual-scene responses, EOG signals were first preprocessed and then their complexity was characterized using sample, fuzzy, and permutation entropy.

### 3.1 Emotion-dependent changes in EOG signal complexity

**Figure 2:**
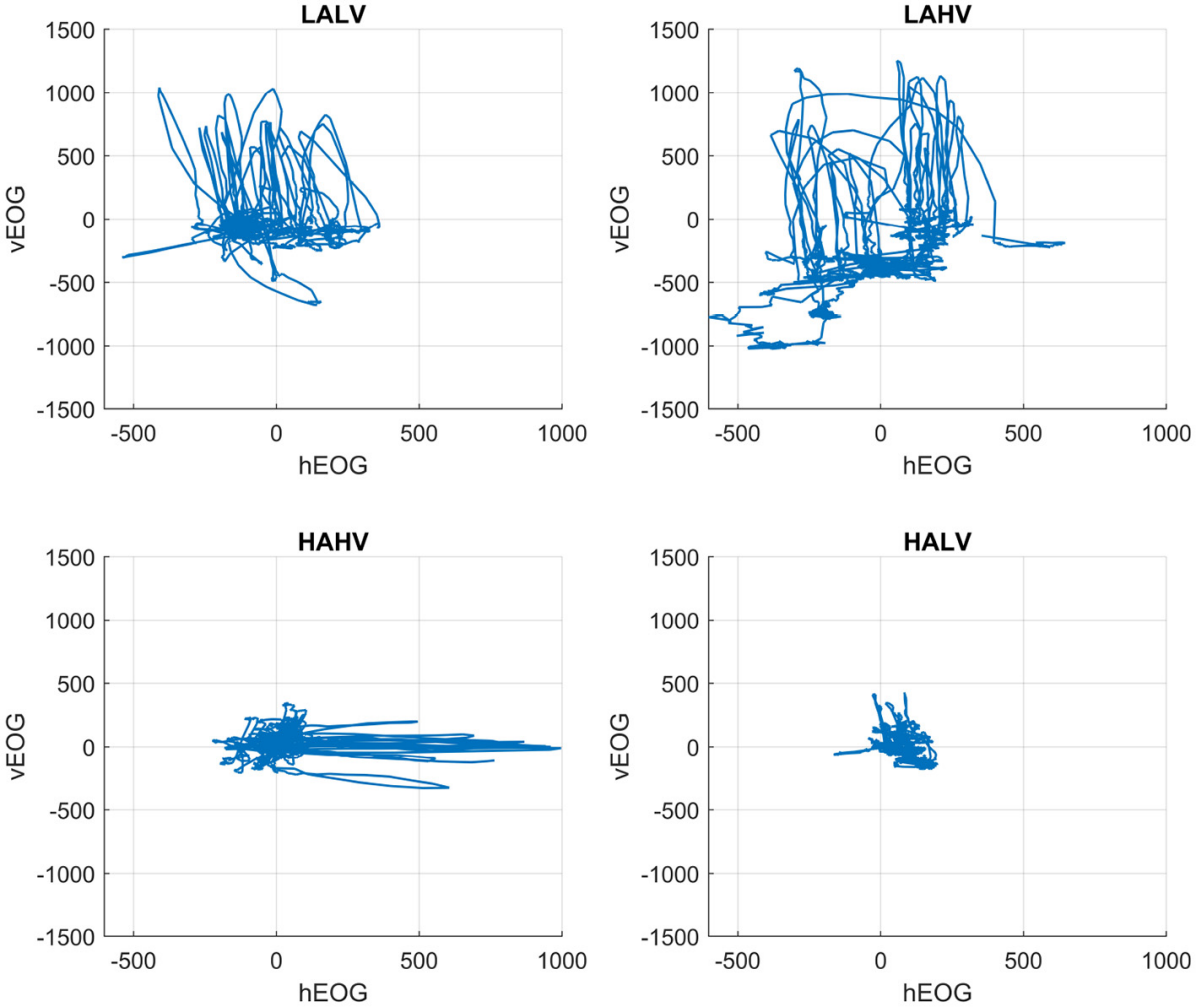
Eye-movement trajectories by emotion. Horizontal (hEOG) vs. vertical (vEOG) potentials (μ*V*) plotted over one minute for LALV, LAHV, HAHV, and HALV. LAHV shows the broadest spread; LALV is moderate; HAHV is elongated predominantly along the horizontal axis; HALV remains tightly clustered.

Distinct oculomotor patterns emerged when horizontal vs. vertical EOG trajectories were plotted over the full one-minute duration of representative video clips for each emotion (Figure 2). LAHV showed the widest spatial spread, LALV was intermediate, HAHV traces were elongated predominantly along the horizontal axis, and HALV remained tightly clustered around the center. These qualitative differences suggest systematic modulation of eye-movement dynamics across affective dimensions.

**Figure 3:**
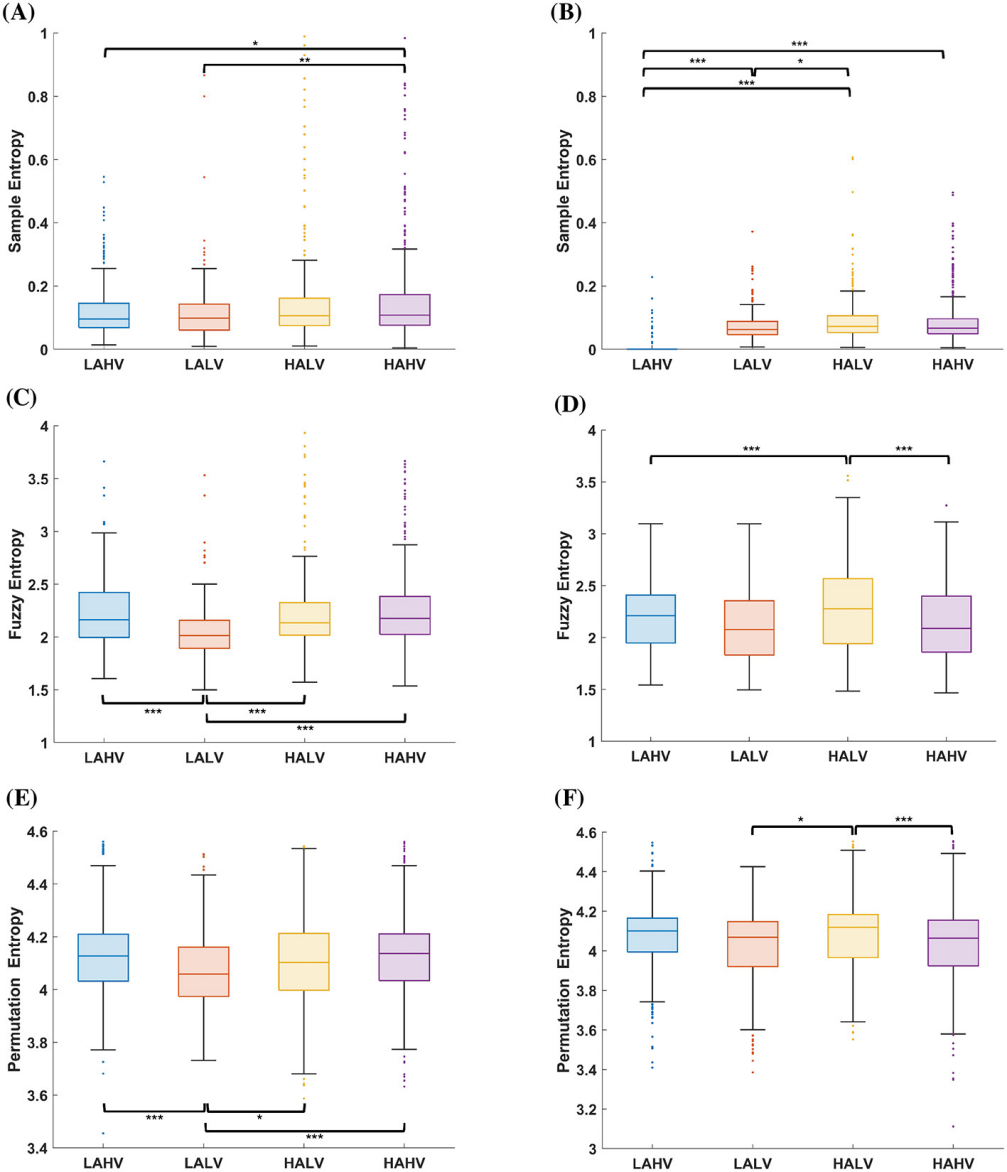
Entropy measures of EOG signals. **(A, B)** Sample entropy, **(C, D)** fuzzy entropy, and **(E, F)** permutation entropy for horizontal (left column) and vertical (right column) EOG channels across the four affective conditions (LAHV, LALV, HAHV, and HALV). Kruskal–Wallis tests revealed significant overall differences between conditions (*p* < 0.005). Significant *post-hoc* pairwise comparisons after correction (Dunn–Šidák, *p* < 0.05) are marked by brackets, with *p*-values shown above each comparison (significance: *p < 0.05, **p < 0.01, ***p < 0.001).

To verify that these differences reflect emotion-related eye movement behavior beyond what would be expected from simple responses to scene content, the signal complexity was quantified using sample, fuzzy, and permutation entropy for each EOG channel and emotion (Figure 3). A Kruskal–Wallis test revealed significant overall differences across the four affective states for all three entropy measures in both hEOG and vEOG (*p* < 0.005). However, effect size analysis revealed a marked divergence between entropy measures: sample entropy showed a large effect (ε^2^ = 0.46), whereas FuzzyEn (ε^2^ = 0.02) and permutation entropy (ε^2^ = 0.005) exhibited only small to negligible effects. *Post hoc* pairwise comparisons were conducted using Dunn tests with Šidák correction.

For hEOG, the sample entropy (Figure 3A) was significantly lower in low-arousal states compared to high-arousal states (HAHV > LAHV, *p* = 0.0299; HAHV > LALV, *p* = 0.0069), indicating reduced horizontal eye-movement variability under lower arousal. Similarly, FuzzyEn (Figure 3C) exhibited significantly lower values in LALV states compared to HAHV, HALV, and LAHV (*p* < 1 × 10^−7^). Likewise, the permutation entropy (Figure 3E) was lower in LALV than in HAHV, HALV, and LAHV (*p* = 6.82 × 10^−6^; *p* = 0.041; *p* = 4.84 × 10^−5^, respectively). Despite statistical significance, interquartile ranges showed notable overlap across affective states, indicating that observed differences represent modest shifts in signal complexity rather than complete separation between conditions.

For vEOG, the sample entropy (Figure 3B) was significantly lower in LAHV than in HAHV, HALV, and LALV (*p* < 0.001), suggesting more regular and constrained vertical eye-movement dynamics under low arousal. Additionally, under low-valence conditions, LALV showed lower entropy than HALV (*p* = 0.0482), consistent with an arousal-dependent reduction in vEOG complexity. FuzzyEn (Figure 3D) was higher in HALV than in LAHV and HAHV (*p* < 5 × 10^−5^). Although visual inspection of interquartile ranges suggests partial overlap between conditions, only comparisons surviving Dunn–Šidák correction are reported as significant. The HALV–LALV contrast did not reach corrected significance despite similar dispersion patterns. Permutation entropy (Figure 3F) showed a similar pattern with values higher in HALV than LALV and HAHV (*p* < 0.02). Although sample entropy approached low values in the LAHV condition, this does not imply that the signal was constant or strictly periodic. Reduced amplitude variance can substantially lower sample entropy when the tolerance parameter is proportional to the signal standard deviation. In contrast, permutation entropy is based on ordinal ranking of successive samples and is invariant to amplitude scaling; therefore, it need not decrease when overall variance is reduced. The two measures thus capture complementary aspects of temporal structure rather than redundant properties of the signal.

To better dissociate arousal and valence contributions, comparisons were examined within the same arousal level across valence conditions and vice versa. Within high-arousal states (HAHV vs. HALV), entropy differences were modest relative to arousal-driven contrasts. In contrast, comparisons between high- and low-arousal conditions within the same valence level (e.g., HAHV vs. LAHV; HALV vs. LALV) consistently revealed higher entropy under high arousal. This pattern suggests that arousal contributes more strongly to EOG complexity than valence alone.

Because entropy was computed directly from EOG signals, these differences may partly reflect stimulus-driven properties such as motion dynamics inherent to the video clips. However, the structured and dimension-specific patterns observed across arousal contrasts indicate that oculomotor variability is systematically modulated during affective stimulation rather than reflecting purely random scene characteristics. These findings motivate further examination of EOG dynamics within the subsequent brain–eye coupling analysis.

### 3.2 Emotion-dependent changes in brain–eye interactions

**Figure 4:**
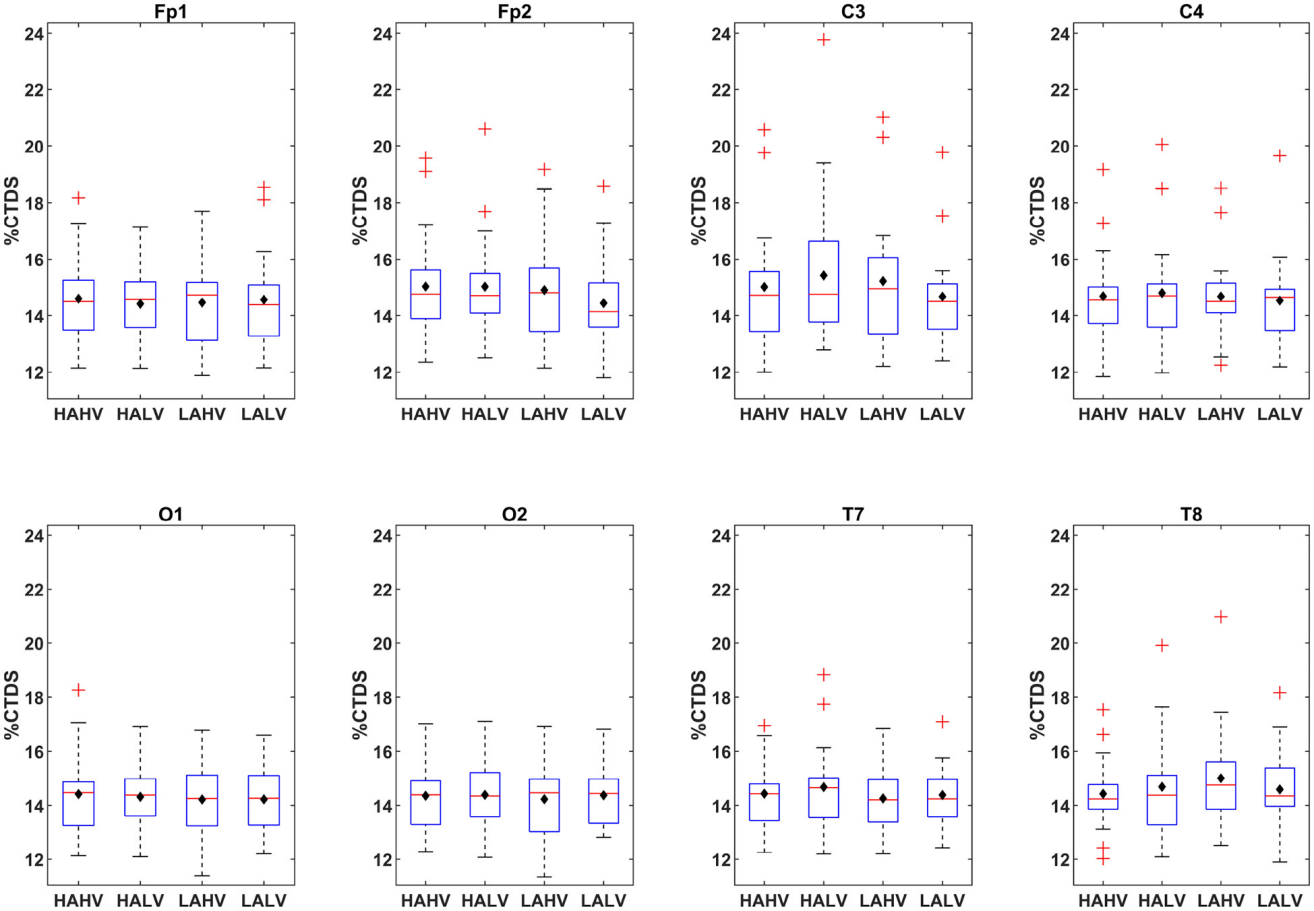
Average interaction-strength distributions by electrode across affective states. Box plots of channel-wise %CTDS link strengths for each electrode under the four affective states (HAHV, HALV, LAHV, and LALV). Boxes represent the interquartile range with the median (red line), and whiskers extend to 1.5IQR, after which red crosses denote outliers, which in this case reflect stronger than average interactions. Black diamonds denote the mean.

**Figure 5:**
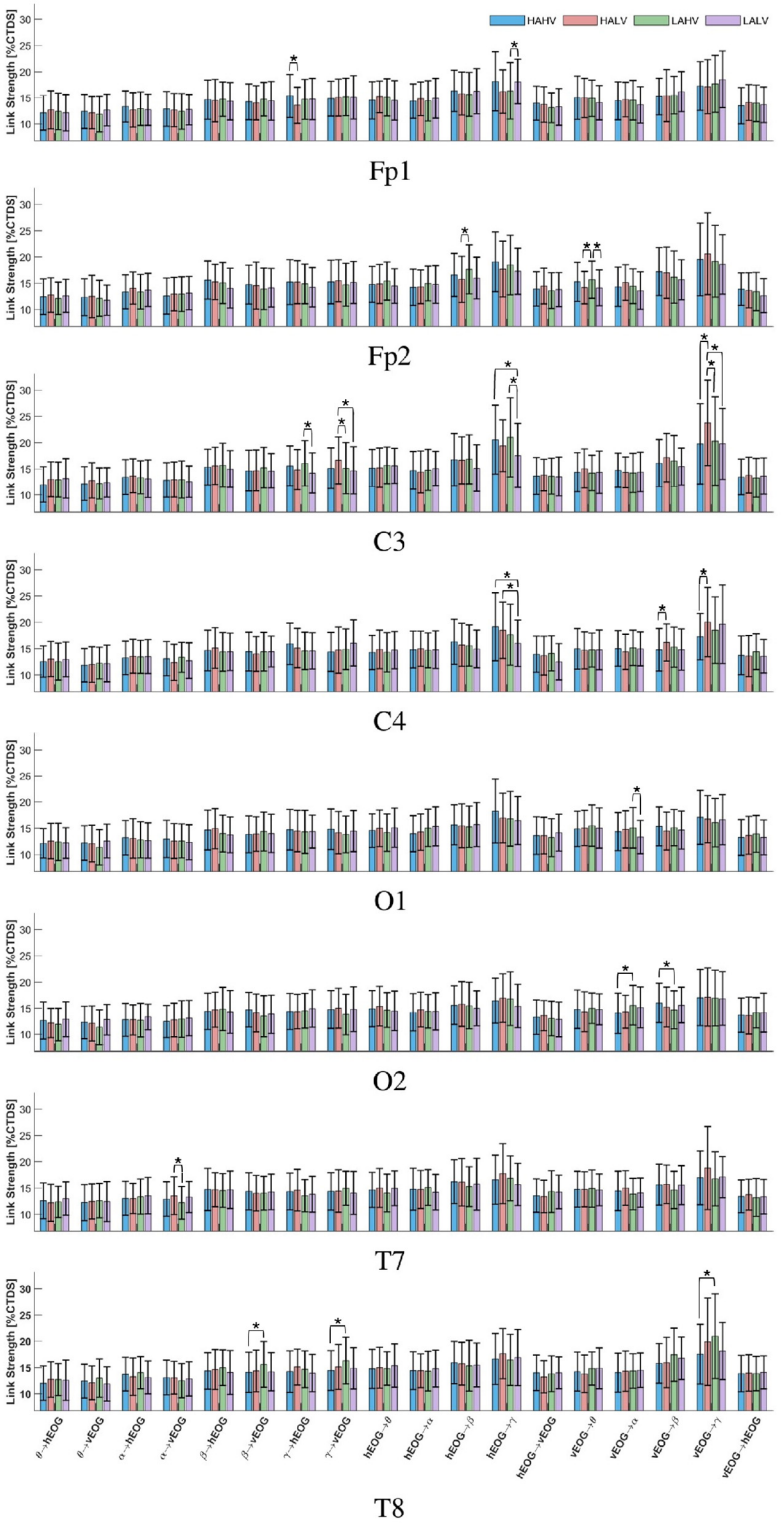
Electrode-wise EEG–EOG directed interaction strengths (%CTDS) across affective states. Each panel corresponds to one EEG electrode. Within each panel, bar groups represent the selected directed EEG–EOG interaction pairs, and the four bars within each group correspond to the affective conditions HAHV, HALV, LAHV, and LALV. Each bar shows the mean %CTDS coupling strength estimated using the CTDS method for interactions between EEG rhythms (θ, α, β, γ) and ocular signals (hEOG, vEOG), including both eye → brain and brain → eye pathways. Error bars denote standard deviation across samples. Bars marked with * indicate statistically significant differences between affective states (*post hoc* Dunn-Šidák test, *p* < 0.05; see Table 3).

To determine whether affective states modulate brain–eye coordination, bidirectional EEG–EOG coupling was examined across cortical regions and frequency bands (Figure 4). The boxplots illustrate the distribution and central tendency of the averaged coupling strength measure (%CTDS) for bidirectional EEG–EOG interactions across electrodes and affective states, highlighting their spatial organization. Across electrodes, coupling values were generally concentrated between approximately 12 and 20% CTDS, with noticeable differences in variability across regions. Sensorimotor and frontal sites exhibited descriptively broader interquartile ranges and somewhat higher medians than temporal and occipital regions, suggesting relatively stronger and more variable interactions in central cortical areas. In particular, C3 appeared to show the widest dispersion across affective states, indicating greater variability in left sensorimotor coupling. In contrast, occipital sites (O1, O2) displayed narrower distributions and lower medians, reflecting comparatively weaker interactions. A modest right-hemisphere prominence was observed at the frontal, temporal, and occipital sites, whereas left sensorimotor coupling appeared slightly stronger. Bar charts of the group-averaged strengths of the studied EEG–EOG interactions at each electrode (Figure 5) show a pronounced γ-band peak in the EOG → EEG direction. EEG → EOG coupling also increased with frequency and reached its maximum in the γ band across most electrodes. To statistically examine individual interactions, the Kruskal–Wallis test was conducted for each directed interaction in the four emotional states (Table 1), revealing that most of the emotion effects were observed in the gamma band. EOG → EEG gamma interactions at Fp1, C3, C4, and T8 and EEG → EOG gamma interactions at Fp1, C3, C4, and T8 differed between affective states. Lower- and mid-frequency effects were limited to isolated electrodes rather than appearing consistently across multiple sites. Although the interaction between hEOG and vEOG within the C4 channel-specific model showed significant differences between affective states, no *post hoc* comparisons remained significant after correction. Across all significant tests, effect sizes were small (ε^2^≈0.01 − 0.03), showing that emotion-related differences in coupling strength were limited in magnitude (Table 2). Such small but consistent effects are expected given the distributed nature of physiological coordination in complex affective states, reflecting subtle network-level modulation rather than large localized changes characteristic of major physiological shifts. Dunn *post hoc* pairwise comparisons applying the Šidák correction showed that emotion-dependent modulations were mainly driven by γ (Table 3). C3 resulted in the densest cluster of emotion-sensitive gamma interactions in both directions, whereas C4 showed significant modulations in unidirectional EOG → EEG interactions that extended into both the β and γ EEG bands. At Fp1, bidirectional interaction between hEOG and the γ-band EEG distinguished valence. At Fp2, the vEOG → θ differed for LAHV vs. LALV and for HALV vs. LAHV, and hEOG → β also distinguished HALV from LAHV. In temporal electrodes, significant comparisons involved vEOG exclusively, and most of the interactions were from EEG to EOG. At T7, the interaction α → vEOG separated HALV from LAHV. At T8, three interactions were significantly different between HAHV and LAHV: two brain-to-eye interactions, β → vEOG and γ → vEOG, and one eye-to-brain interaction vEOG → γ.

**Table 1:** Kruskal–Wallis test resultant *p*-values for comparisons of interaction strength between affective states.

Interaction	Fp1	Fp2	C3	C4	O1	O2	T7	T8
θ → hEOG	0.5385	0.6126	0.0331^*^	0.7451	0.7722	0.1315	0.5554	0.2216
θ → vEOG	0.4864	0.6902	0.3273	0.6637	0.1796	0.0756	0.8665	0.1668
α → hEOG	0.2081	0.4236	0.7390	0.9202	0.4562	0.6197	0.5434	0.2846
α → vEOG	0.7955	0.5860	0.6686	0.2575	0.5701	0.3863	0.0229^*^	0.4698
β → hEOG	0.7934	0.0667	0.8012	0.5001	0.1955	0.7836	0.9834	0.3275
β → vEOG	0.4104	0.3663	0.5988	0.8052	0.7434	0.0715	0.6085	0.0471^*^
γ → hEOG	0.0050^*^	0.4875	0.0314^*^	0.0511	0.8908	0.7434	0.0932	0.1420
γ → vEOG	0.9877	0.6440	0.0085^*^	0.0871	0.2155	0.1937	0.3732	0.0159^*^
hEOG → θ	0.3332	0.4340	0.7599	0.5634	0.3845	0.5775	0.2211	0.7910
hEOG → α	0.6474	0.3696	0.8303	0.9301	0.0392^*^	0.7454	0.2961	0.6915
hEOG → β	0.4579	0.0305^*^	0.0684	0.2124	0.8886	0.8597	0.4514	0.7798
hEOG → γ	0.0045^*^	0.1580	0.0087^*^	0.0071^*^	0.1544	0.2250	0.2114	0.4352
hEOG → vEOG	0.1063	0.4992	0.9907	0.0438^*^	0.5540	0.5716	0.1180	0.3377
vEOG → θ	0.3763	0.0052^*^	0.2095	0.9757	0.4553	0.6589	0.9071	0.3299
vEOG → α	0.3853	0.0729	0.6625	0.2457	0.0182^*^	0.0208^*^	0.1054	0.6496
vEOG → β	0.4597	0.0584	0.1884	0.0282^*^	0.2493	0.0339^*^	0.1420	0.0522
vEOG → γ	0.4549	0.4348	0.0005^*^	0.0341^*^	0.5986	0.9826	0.4915	0.0187^*^
vEOG → hEOG	0.5518	0.0857	0.7654	0.3851	0.4344	0.5462	0.7344	0.9975

^*^indicates a significant difference in interaction strength between affective states (*p* < 0.05).

**Table 2:** Epsilon-squared (ε^2^) effect sizes for directional EEG–EOG interactions across electrodes.

Interaction	Fp1	Fp2	C3	C4	O1	O2	T7	T8
θ → hEOG	–	–	0.0133	–	–	–	–	–
θ → vEOG	–	–	–	–	–	–	–	–
α → hEOG	–	–	–	–	–	–	–	–
α → vEOG	–	–	–	–	–	–	0.0154	–
β → hEOG	–	–	–	–	–	–	–	–
β → vEOG	–	–	–	–	–	–	–	0.0117
γ → hEOG	0.0221	–	0.0136	–	–	–	–	–
γ → vEOG	–	–	0.0202	–	–	–	–	0.0173
hEOG → θ	–	–	–	–	–	–	–	–
hEOG → α	–	–	–	–	0.0126	–	–	–
hEOG → β	–	0.0141	–	–	–	–	–	–
hEOG → γ	0.0226	–	0.0201	0.0218	–	–	–	–
hEOG → vEOG	–	–	–	0.0123	–	–	–	–
vEOG → θ	–	0.0233	–	–	–	–	–	–
vEOG → α	–	–	–	–	0.0166	0.0159	–	–
vEOG → β	–	–	–	0.0146	–	0.0133	–	0.0112
vEOG → γ	–	–	0.0339	0.0136	–	–	–	0.0165
vEOG → hEOG	–	–	–	–	–	–	–	–

Values represent ε^2^ effect sizes. Dashes (–) indicate non-significant interactions (*p*≥0.05).

**Table 3:** *Post hoc* Dunn–Šidák comparisons of significantly different directional EEG–EOG interactions between affective states.

Electrode	Interaction	Comparison	Corrected *p*-value
Fp1	γ → hEOG	HAHV vs. HALV	0.0024
	hEOG → γ	LAHV vs. LALV	0.0461
Fp2	hEOG → β	HALV vs. LAHV	0.0296
	vEOG → θ	HALV vs. LAHV	0.0395
		LAHV vs. LALV	0.0425
C3	γ → hEOG	LAHV vs. LALV	0.0499
	γ → vEOG	HALV vs. LAHV	0.0342
		HALV vs. LALV	0.0187
	hEOG → γ	HAHV vs. LALV	0.0103
		LAHV vs. LALV	0.0125
	vEOG → γ	HAHV vs. HALV	0.0005
		HALV vs. LAHV	0.0081
		HALV vs. LALV	0.0261
C4	hEOG → γ	HAHV vs. LALV	0.0057
		HALV vs. LALV	0.0363
	vEOG → β	HAHV vs. HALV	0.0326
	vEOG → γ	HAHV vs. HALV	0.0285
O1	vEOG → α	LAHV vs. LALV	0.0169
O2	vEOG → α	HAHV vs. LAHV	0.0340
	vEOG → β	HAHV vs. LAHV	0.0296
T7	α → vEOG	HALV vs. LAHV	0.0194
T8	β → vEOG	HAHV vs. LAHV	0.0351
	γ → vEOG	HAHV vs. LAHV	0.0081
	vEOG → γ	HAHV vs. LAHV	0.0152

Overall, most of the significant coupling differences arose when the two states that were compared differed in only one affective dimension (HAHV vs. HALV, LAHV vs. LALV, HAHV vs. LAHV, HALV vs. LALV). Comparisons in which the affective states are on the diagonal from each other on the affect complex showed fewer effects (HALV vs. LAHV and HAHV vs. LALV).

## 4 Discussion

This study demonstrates that the complexity of eye movements and their directional interactions with EEG rhythms are sensitive to emotional context, with changes in EOG–EEG coupling varying across affective states. Entropy analyses revealed a selective pattern of effects: sample entropy showed robust modulation across affective conditions (ε^2^ = 0.46), whereas fuzzy and permutation entropy exhibited only small to negligible effects (ε^2^ = 0.02 and ε^2^ = 0.005, respectively). Sample, fuzzy, and permutation entropy values were generally lower in low-arousal or low-valence conditions, suggesting that eye movements become more stereotyped under reduced emotional intensity or unpleasant affect. This trend complements prior evidence associating higher entropy with greater emotional engagement (Lee et al., 2020). The stronger effect observed for sample entropy suggests that affect-related modulation of EOG complexity may depend on the specific mathematical formulation of entropy, rather than reflecting a uniform change across all irregularity metrics. Together, these findings indicate that EOG captures structured, emotion-related behavioral complexity rather than merely reflecting scene-driven fluctuations. Consistent with this interpretation, a supplementary control analysis revealed small but statistically significant inverse associations between trial-wise EOG sample entropy and mean heart rate derived from PPG (Supplementary Table S3, Supplementary Figure S1). These results provide preliminary convergent validity between ocular complexity and an independent autonomic arousal index.

CTDS analysis showed descriptively higher interaction strengths at sensorimotor and frontal electrodes compared to temporal and occipital sites (Figure 5), suggesting a possible spatial organization that may reflect structural and functional pathways linking oculomotor and frontal/parietal regions (de Schotten et al., 2012; Budisavljevic et al., 2017). The descriptively stronger coupling observed at some right-hemisphere sites is consistent with the well-established dominance of the right hemisphere for emotional visual attention (Hartikainen, 2021). Across the analyzed electrodes, most significant emotion-related changes in EEG–EOG interactions occurred in the gamma band. Right-hemisphere involvement was particularly evident in the gamma-band interactions, especially at the right temporal electrode (T8) (Table 1). While gamma effects were present bilaterally, their prominence at T8 aligns with the established role of the right hemisphere in emotional attention and vigilance. Gamma oscillations have been known to play a role in attention, visual information processing, and perception of audiovisual speech (Kaiser et al., 2005; Müller et al., 2000; Lin et al., 2015). The present study extends this by showing that gamma-mediated EEG–EOG interactions appeared modulated across affective states during audiovisual viewing. Importantly, gamma-band oscillations are not exclusively linked to emotional processing and are widely associated with attentional engagement and perceptual demands. Thus, part of the observed gamma-mediated EEG–EOG interactions may reflect stimulus-driven attentional modulation during audiovisual viewing. However, the modulation patterns were structured across affective dimensions rather than showing uniform increases across conditions. Coupling differences were most prominent when states differed along a single dimension (arousal or valence), suggesting that the effects cannot be explained solely by generalized attentional load. Instead, the findings likely reflect coordinated brain–eye dynamics during affective stimulation, integrating emotional and attentional processes within a unified network response. From a methodological standpoint, gamma-band EEG signals can include saccadic artifacts. Although ICA was applied during preprocessing to remove ocular activity, and gamma effects showed spatial and directional specificity, artifactual high-frequency contributions cannot be entirely excluded. However, the observed gamma-band EEG–EOG interactions were not restricted to frontal electrodes, but were also prominently expressed in central sensorimotor and temporal channels. The spatial specificity and bidirectional organization of the effects therefore argue against a purely artifactual origin.

Eye-to-brain interactions were more frequently observed at frontal and occipital electrodes, whereas significant brain-to-eye interactions were noted at C3, T7, and T8. These patterns suggest a potential asymmetry in directional coupling. This asymmetry is matched by hierarchical accounts of vision where an initial feed-forward sweep is modulated by top-down influences (Delorme et al., 2004). When two stimuli follow each other in rapid succession, interference between the feedback generated by the first and the feed-forward signal of the second can alter behavioral accuracy, underlining the functional independence of the two directions (Martin et al., 2019). Reviews of cortical circuitry also emphasize that ascending and descending projections serve distinct but complementary roles (Kafaligonul et al., 2015). Descending cortical commands converge on the cranial nerves III, IV, and VI that control eye movements. Because these effector pathways react within a few hundred milliseconds, EOG recordings can offer a rapid read-out of changes in emotion, arousal, and cognitive load (Sanders, 2009). Smooth-pursuit slowing, saccadic dyscontrol, vergence errors, and abnormal pupillary dynamics reported in schizophrenia, mood and anxiety disorders, and obsessive–compulsive disorder all appear to reflect altered feedback along this pathway (Feinberg and Rosner, 2020; Reuter and Kathmann, 2004; Lencer et al., 2004; Sanders, 2009). Therefore, quantifying EEG–EOG interactions is a promising option to identify mechanistic markers of affective states. Prior work suggests that eye dynamics are modulated by cognitive engagement, indicating top-down influences on ocular behavior. The same study also reported that slow heart rate fluctuations precede pupil changes, suggesting a bottom-up autonomic contribution to ocular and cortical arousal (Madsen and Parra, 2024). The present CTDS pattern supports a bidirectional model in which bottom-up sensory inflow drives rapid appraisal, and more selective top-down signals adjust eye position in line with emotional context. This organization aligns with the predictive coding theory of brain function, a neurophysiological framework proposing that higher-order regions generate expectations and compare them with sensory input to identify mismatches between prediction and outcome (Rauss and Pourtois, 2013). In this context, the observed eye-to-brain interactions are consistent with ascending sensory signals carrying novel visual information, while brain-to-eye feedback may reflect descending modulatory influences that adjust ocular activity in line with contextual demands. The pattern also agrees with previous network physiology studies emphasizing reciprocal integration between cortical and peripheral systems during cognitive attention, alertness, and emotional processing (Marzbanrad et al., 2020; Alskafi et al., 2024; Candia-Rivera et al., 2024). Recent accounts further highlight that visual and interoceptive systems interact within hierarchical predictive models that align exteroceptive and visceral signals to sustain adaptive perception and emotional awareness (Candia-Rivera et al., 2024).

These results suggest that both ocular complexity and directed EEG–EOG interactions vary with affective state and may serve as markers of coordinated sensory-cortical activity during emotional processing. The small effect sizes likely reflect distributed physiological coordination, in which multiple systems jointly contribute to regulation. In complex affective states, subtle yet consistent changes are expected, as adaptive tuning across interconnected systems supports network-level regulation rather than localized responses. Emotional demands may therefore gradually adjust coupling across interconnected systems instead of producing large isolated effects. Therefore, these features could possibly be used to objectively measure emotion-related changes in audiovisual processing. Because these features can be derived from single scalp EEG electrodes and EOG channels, and portable EEG/EOG headsets are becoming widely available (Casson, 2019; He et al., 2023; Belkhiria et al., 2022), they are suited for use in biofeedback systems and in emotion-aware interfaces that automatically adjust to the implicit signals of the user rather than to their feedback (Miraz et al., 2021). Furthermore, the results support a bottom-up view where changing activity, for example through visual stimulation, can be rechanneled into the same circuits that influence mood, providing a practical route by which peripheral interventions may assist in monitoring and relieving affective disorders (Mizumoto et al., 2024; Canbeyli, 2010; Porges, 2022).

## 5 Limitations and future directions

This work provides an initial overview of how brain rhythms and eye movements interact during emotional viewing, but several aspects of the experimental design limit the generalizability of the findings. First, this study used the DEAP dataset, which contains recordings from 32 healthy European university students who viewed short music video clips on a flat screen; the small, demographically narrow sample and artificial task limit the generalizability to everyday social settings. Investigating the same interactions in virtual reality scenes or live interactions would indicate whether the pattern is retained in more natural conditions. Second, scalp EEG recordings provide only an approximate location of where signals originate. Third, the analysis paired the EOG with only eight scalp sites; alternative pathways may therefore have gone undetected. Future work could combine dense or source-localized EEG to better characterize the spatial origins of the observed coupling patterns. Fourth, an additional methodological limitation concerns the frequency characteristics of the EOG preprocessing. EOG signals were low-pass filtered at 15 Hz, which removes high-frequency components associated with microsaccades (approximately 20–40 Hz). Microsaccadic spike potentials are known to modulate scalp gamma activity and may influence high-frequency EEG–EOG coupling estimates. Although ICA-based correction was applied to remove ocular artifacts and gamma-band effects showed spatial and directional specificity, the absence of simultaneous high-resolution eye tracking prevents definitive separation of neural gamma synchrony from residual miniature saccadic potentials. Future studies combining CTDS analysis with concurrent eye tracking would allow more precise dissociation of neural and oculomotor high-frequency contributions. Fifth, although the emotions were rated by each participant, the data were analyzed only at the group level. Finally, the CTDS framework used here quantifies interactions between a single EEG rhythm and a single EOG signal at a time, using partial correlations to control for the other signals; even so, the approach remains pairwise and may not fully capture the simultaneous interplay between all brain rhythms and eye movement measures. In addition, CTDS relies on higher-order partial cross-correlation and therefore primarily captures linear dependencies between signals. Non-linear interactions between EEG rhythms and oculomotor dynamics may not be fully characterized within this framework. Consequently, the small effect sizes observed in some interactions may reflect both distributed physiological modulation and methodological constraints inherent to linear coupling estimators. Incorporating non-linear interaction metrics may provide complementary insight into brain–eye coordination. Future research could expand on these findings by examining their applicability in clinical and digital health contexts, particularly for assessing emotional regulation and cognitive load.

## 6 Conclusion

To our knowledge, this study is among the first to quantify directional, frequency-specific EEG–EOG coupling during affective audiovisual stimulation using higher-order partial cross-correlation with time delay stability constraints. Using this framework, we show that emotional state alters both the complexity of eye movements and their directed interaction with cortical oscillations. Gamma-band interactions emerged as the most sensitive marker of brain–eye coupling, implying that fast neural rhythms help coordinate gaze during affective processing. Because EOG carries emotion-relevant information rather than being a disposable artifact, it deserves a place in future multimodal studies. The CTDS framework offers a practical tool for measuring these interactions and could support adaptive interfaces and biofeedback applications.

## Data Availability

The original contributions presented in the study are included in the article/Supplementary material, further inquiries can be directed to the corresponding author.
