# Supplementary material for: Quantifying emotion-dependent brain–eye interactions during audiovisual emotional stimulation
Source: Front Syst Neurosci. 2026 Apr 15;20:1744042. doi: 10.3389/fnsys.2026.1744042 (PMC13124692; doi:10.3389/fnsys.2026.1744042)
Supplement: Supplementary file 1 [file Data_Sheet_1.pdf]

Supplementary Material

1 CHARACTERISTICS OF PARTICIPANTS IN THE DEAP DATASET

Table S1. Summary of demographic and lifestyle characteristics of study participants. Counts do not always sum to the total number of participants (N=32) because some survey responses were missing or not reported in the original dataset.

Variable	Category / Statistic	Value / Count
Age	Min–Max	19 - 37
	Mean	27.1
	Median (IQR)	27 (24 - 30.75)
Gender	Male	17
	Female	15
Handedness	Right	31
	Left	1
Vision	Normal	14
	Corrected to normal	15
	Acceptable uncorrected	2
Education	MA/MSc/Ir	17
	BA/BSc/Ing	10
	High school	2
	PhD	2
Alcohol consumption	Regularly	21
	Never	10
Coffee consumption	Consumes coffee	17
	Does not consume coffee	14
Tea consumption	Consumes tea	21
	Does not consume tea	10
Tobacco consumption	Never	29
	Regularly	2
Other medication consumption	Never	23
	Any use	6
Level of Alertness	Medium	25
	High	5
	Low	1

2 MUSIC-VIDEO STIMULI AND ONLINE SUBJECTIVE RATINGS

Table S2: Music-video stimuli used in the physiological experiment, with valence and arousal ratings obtained during the independent online subjective annotation phase described in the DEAP dataset. Each clip was rated by N volunteers on 9-point self-assessment manikin scales for valence and arousal. Values reported here represent mean ± standard deviation of the online ratings. The highlight start indicates the beginning (in seconds) of the 60-second excerpt presented during the experiment.

Artist	Title	Start (s)	N	Valence (M±SD)	Arousal (M±SD)
Emilíana Torrini	Jungle Drum	81	14	6.86 ± 1.30	5.86 ± 2.20
Lustra	Scotty Doesn't Know	141	14	5.93 ± 2.05	6.93 ± 1.98
Michael Franti & Spearhead	Say Hey (I Love You)	151	14	7.14 ± 1.19	4.86 ± 1.46
Jackson 5	Blame It On The Boogie	21	15	6.93 ± 2.32	6.47 ± 1.93
Grand Archives	Miniature Birds	151	14	5.93 ± 1.79	3.36 ± 1.34
Bright Eyes	First Day Of My Life	6	14	6.57 ± 1.40	4.21 ± 2.51
Jason Mraz	I'm Yours	6	15	7.07 ± 1.44	4.73 ± 2.11
Bishop Allen	Butterfly Nets	6	15	6.47 ± 1.36	4.00 ± 1.79
Porcupine Tree	Normal	126	15	4.20 ± 1.42	3.73 ± 1.81
Wilco	How To Fight Loneliness	181	15	3.33 ± 1.19	4.47 ± 2.00
The Submarines	Darkest Things	41	15	5.13 ± 1.09	2.40 ± 1.62
James Blunt	Goodbye My Lover	186	15	3.33 ± 1.35	2.93 ± 1.69
A Fine Frenzy	Goodbye My Almost Lover	211	15	4.20 ± 1.64	3.60 ± 1.20
Kings Of Convenience	The Weight Of My Words	191	15	4.20 ± 1.80	3.00 ± 1.51
Mortemia	The One I Once Was	211	15	3.67 ± 1.49	5.47 ± 2.06
Marilyn Manson	The Beautiful People	6	15	4.67 ± 1.49	6.40 ± 1.93
Dead To Fall	Bastard Set Of Dreams	126	15	3.93 ± 2.05	6.13 ± 2.09

Table S1 continued

Artist	Title	Start (s)	N	Valence (M±SD)	Arousal (M±SD)
The B52's	Love Shack	181	15	7.00 ± 1.71	5.93 ± 1.98
Blur	Song 2	6	15	7.20 ± 1.76	7.33 ± 1.58
Blink 182	First Date	51	15	6.13 ± 1.50	6.20 ± 1.60
Benny Benassi	Satisfaction	26	15	6.67 ± 1.89	6.47 ± 2.06
Air	Moon Safari	211	15	6.07 ± 1.61	3.00 ± 1.51
Louis Armstrong	What A Wonderful World	86	15	7.13 ± 1.41	3.87 ± 1.86
Manu Chao	Me Gustas Tu	46	15	7.53 ± 1.26	4.47 ± 1.59
Lily Allen	Fuck You	6	15	7.27 ± 1.34	6.07 ± 1.77
Taylor Swift	Love Story	151	15	6.27 ± 1.18	4.13 ± 1.71
Queen	I Want To Break Free	6	15	7.07 ± 1.84	6.40 ± 1.99
Madonna	Rain	31	15	4.33 ± 2.02	3.13 ± 1.78
Sia	Breathe Me	6	16	3.25 ± 1.30	2.75 ± 1.44
Christina Aguilera	Hurt	181	16	3.44 ± 1.32	3.63 ± 1.83
Enya	May It Be (Saving Private Ryan)	86	15	3.20 ± 1.76	3.67 ± 1.89
DJ Paul Elstak	A Hardcore State Of Mind	131	14	4.79 ± 1.93	6.36 ± 1.95
Diamanda Galas	Gloomy Sunday	41	14	4.14 ± 1.81	4.21 ± 1.52
Napalm Death	Procrastination On The Empty Vessel	41	15	3.53 ± 1.89	6.33 ± 2.12
Sepultura	Refuse Resist	6	15	4.93 ± 2.29	7.27 ± 1.34
Cradle Of Filth	Scorched Earth Erotica	6	15	3.27 ± 1.61	5.87 ± 2.28
Gorgoroth	Carving A Giant	166	15	3.27 ± 2.11	5.33 ± 2.24
Dark Funeral	My Funeral	251	15	3.47 ± 2.33	5.33 ± 2.39
Rage Against The Machine	Bombtrack	6	15	5.87 ± 2.19	7.07 ± 1.44
Arch Enemy	My Apocalypse	201	15	3.73 ± 2.59	5.73 ± 2.24

3 CONVERGENT VALIDITY ANALYSIS: ASSOCIATION BETWEEN EOG ENTROPY AND HEART RATE

To examine the convergent validity of EOG complexity as an index of arousal-related physiology, we computed trial-wise mean heart rate (HR, derived from PPG) and correlated it with single-trial sample entropy of horizontal and vertical EOG across all participants and stimuli. Spearman correlations (Table S3) indicated small but systematic negative associations between HR and EOG entropy, which are illustrated in Supplementary Figure S1.

Table S3. Spearman correlations between trial-wise EOG sample entropy and mean HR across all participants and music-video trials.

**Measure**	**Spearman ρ**	**p-value**
vEOG Sample Entropy vs HR	-0.176	2.40×10^{-10}
hEOG Sample Entropy vs HR	-0.180	9.61×10^{-11}

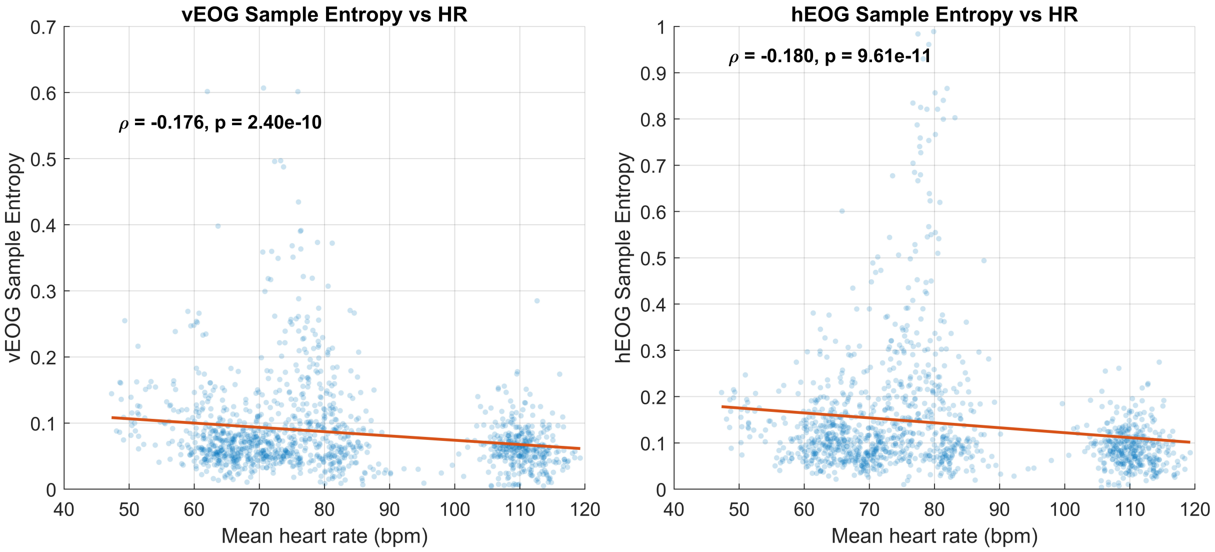

Figure S1. Trial-wise relationship between EOG sample entropy and mean HR. Left panel: vEOG sample entropy vs HR. Right panel: hEOG sample entropy vs HR. Each point represents a single participant–trial combination. Solid lines indicate least-squares fits for visualization. Spearman correlations revealed small but statistically significant negative associations, indicating that higher heart rate is modestly associated with lower EOG entropy (i.e., more regular eye-movement dynamics).
